# Urinary dysfunction in patients with vascular cognitive impairment

**DOI:** 10.3389/fnagi.2022.1017449

**Published:** 2023-01-18

**Authors:** Panpan Zhao, Guimei Zhang, Yanxin Shen, Yongchun Wang, Lin Shi, Zicheng Wang, Chunxiao Wei, Weijie Zhai, Li Sun

**Affiliations:** Department of Neurology and Neuroscience Center, The First Hospital of Jilin University, Jilin University, Changchun, China

**Keywords:** urinary dysfunction, urinary incontinence, vascular cognitive impairment, white matter lesions, overactive bladder

## Abstract

Vascular cognitive impairment (VCI) is caused by vascular pathologies, with the spectrum of cognitive disorders ranging from subjective cognitive dysfunction to dementia. Particularly among older adults, cognitive impairment is often complicated with urinary dysfunction (UD); some patients may present with UD before cognitive impairment owing to stroke or even when there are white matter hyperintensities on imaging studies. Patients with cognitive impairment often have both language and movement dysfunction, and thus, UD in patients with VCI can often be underdiagnosed and remain untreated. UD has an impact on the quality of life of patients and caregivers, often leading to poor outcomes. Medical history is an important aspect and should be taken from both patients and their caregivers. Clinical assessment including urinalysis, voiding diary, scales on UD and cognitive impairment, post-void residual volume measurement, uroflowmetry, and (video-) urodynamics should be performed according to indication. Although studies on UD with VCI are few, most of them show that an overactive bladder (OAB) is the most common UD type, and urinary incontinence is the most common symptom. Normal urine storage and micturition in a specific environment are complex processes that require a sophisticated neural network. Although there are many studies on the brain–urinary circuit, the specific circuit involving VCI and UD remains unclear. Currently, there is no disease-modifying pharmacological treatment for cognitive impairment, and anti-acetylcholine drugs, which are commonly used to treat OAB, may cause cognitive impairment, leading to a vicious circle. Therefore, it is important to understand the complex interaction between UD and VCI and formulate individualized treatment plans. This review provides an overview of research advances in clinical features, imaging and pathological characteristics, and treatment options of UD in patients with VCI to increase subject awareness, facilitate research, and improve diagnosis and treatment rates.

## 1. Introduction

Vascular cognitive impairment (VCI) is a spectrum of cognitive disorders, ranging from subjective cognitive decline to mild cognitive impairment (MCI) to dementia. VCI is associated with vascular risk factors and heterogeneous cerebrovascular diseases with confounding pathologies and overlapping with vascular and Alzheimer’s disease (AD) characteristics (Gorelick et al., 2011; van der Flier et al., 2018; Rundek et al., 2021). VCI is the second most common cause of dementia (Goodman et al., 2017) and likely the number one facilitating factor in East Asia (Iadecola et al., 2019). Reportedly, in 2018, there were nearly 50 million people with dementia worldwide, with three times as many predicted by 2050, adding approximately $4 trillion to the global health burden (Iadecola et al., 2019). Vascular dementia accounts for at least 20% of all VCI cases (Gorelick et al., 2011). Patients with VCI typically present with impaired processing speed, executive dysfunction, cognitive impairment, depression, dysarthria, gait disturbances, and urinary dysfunction (UD) (van der Flier et al., 2018; Biesbroek and Biessels, 2022). There is no specific treatment for VCI (Shim, 2014); however, a previous study of patients (*n* = 174) prescribed individually tailored interventions (education, non-pharmacologic, pharmacologic) revealed that multidomain interventions may improve cognition (Isaacson et al., 2019). Lifestyle modifications can reduce the rate of cognitive decline (Rundek et al., 2021) and optimize brain health (Sabbagh et al., 2022). Various works suggest that early detection of cognitive decline helps attenuate further progression; therefore, it is critical to implement methods to predict whether cognitive impairment will occur. A retrospective study of outpatients (*n* = 84) with multi-infarct dementia revealed that UD and gait dysfunction may be used as markers for vascular dementia; this study defined UD as incontinence, frequency, and/or urgency (Kotsoris et al., 1987). This definition of UD is consistent with that of overactive bladder (OAB) syndrome, which is characterized by urinary urgency, often accompanied by increased daytime frequency and nocturia, with or without urgency incontinence and occurs in the absence of urinary tract infections or other pathologies (Abrams et al., 2002). After stroke, OAB is the most frequently reported urinary disturbance (Pettersen and Wyller, 2006; Williams et al., 2012). A systematic review highlighted a significant association between nocturia and cognitive dysfunction (Haddad et al., 2020). Urinary incontinence includes any involuntary leakage of urine (Abrams et al., 2002) and is common in older adults (Goepel et al., 2010; Milsom and Gyhagen, 2019). Stress and urgency incontinence are the most common urinary incontinence subtypes, which may result from a variety of disorders (Brown et al., 2006; Lukacz et al., 2017). UD after stroke is associated with cognitive decline (Kotsoris et al., 1987; Chiang et al., 2015), gait impairment (Kotsoris et al., 1987; Korczyn, 2015), depression (Limampai et al., 2017), and overall poor health outcomes (Rana et al., 2021). In addition, imaging studies suggest that UD after stroke is accompanied by the development of intracranial lacunes and white matter lesions (WMLs) (Sakakibara et al., 1999; Poggesi et al., 2008; Wakefield et al., 2010), which are pathological clinical features common to VCI (van der Flier et al., 2018; Biesbroek and Biessels, 2022). One study found that 40%–60% of patients with stroke presented with incontinence upon admission, 25% at discharge, and 15% remained incontinent 1 year later (Thomas et al., 2019). Further, urinary incontinence has a detrimental impact on the quality of life of both patients and their caregivers (Henriksen, 1991). One prospective observational study of patients with acute stroke (*n* = 215) revealed that morbidity and mortality rates during hospitalization and 3 months after discharge were higher among patients with urinary incontinence upon admission than among their non-incontinent counterparts (Gariballa, 2003). Urinary incontinence is associated with decreased home discharge rates among older frailty adults admitted to rehabilitation centers after stroke (Vluggen et al., 2020).

Several reports have focused on the epidemiology of incontinence after stroke (Henriksen, 1991), however, pathogenesis and treatment are less often mentioned (Brittain et al., 1999; Patel et al., 2001; Thomas et al., 2005, 2008, 2019). Indeed, the mechanisms underlying OAB remain unclear. Anticholinergic drugs are effective for OAB but may themselves cause cognitive impairment (Welk et al., 2021). Patients may withhold UD symptoms from physicians owing to embarrassment and misconceptions about prospective treatment options, resulting in low diagnostic rates and less efficient UD treatment outcomes in VCI. Herein, we reviewed lower urinary tract symptoms in patients with VCI by searching for studies using the following keywords: “urinary dysfunction,” “urinary disorder,” “urinary incontinence,” “vascular urinary incontinence,” “overactive bladder,” “lower urinary tract symptom,” “stroke,” “white matter lesions,” “white matter hypertensions,” “vascular cognitive impairment,” “multi-infarct dementia,” and “dementia” on PubMed. Only studies published in English were included. The purpose of this review was to summarize the available evidence supporting the association between UD and VCI, including clinical characteristics, imaging and pathological features, and treatments. This cohesive resource may help facilitate our understanding of pathogenesis, early diagnosis, and accurate treatment of UD/VCI.

## 2. Epidemiology

Urinary dysfunction is a common health condition in older adults (Goepel et al., 2002; Lukacz et al., 2017) as its prevalence increases with age (Goepel et al., 2002, 2010), particularly among stroke survivors, although the estimates vary (Brittain et al., 1998, 1999). Thomas et al. (2019) reported that urinary incontinence affects 40–60% of hospitalized patients with stroke, with 25 and 15% of patients continuing to experience these problems after returning home and 1 year after discharge, respectively. A longitudinal population-based study in Australia reported that more than 80% of first-stroke survivors present with abnormal urinary symptoms, most commonly nocturia, while urinary incontinence affects 43.5 and 37.7% of patients at 3 and 12 months, respectively (Williams et al., 2012). A retrospective study of multi-infarct dementia (*n* = 84 outpatients) suggested that 50% of participants had UD, although predominantly among men (Kotsoris et al., 1987). This may reflect the fact that men are more likely than women to have cerebrovascular diseases.

## 3. Clinical features

According to Vascular Impairment of Cognition Classification Consensus Study guidelines, VCI is classified as mild and major VCI, which is further classified as post-stroke dementia, multi-infarct dementia, subcortical ischemic vascular dementia, and mixed dementia. White matter hyperintensities (WMHs), cerebral atrophy, infarction, and hemorrhage on MRI are gold standard findings for diagnosing VCI (Skrobot et al., 2018; Iadecola et al., 2019). Brain damage associated with VCI is mostly above the pons, which may cause different degrees of OAB (Griffiths et al., 1994; Pettersen and Wyller, 2006; Williams et al., 2012; Panicker et al., 2015; Raju and Linder, 2020).

On urodynamic examination, both dementia and stroke patients showed reduced bladder capacity, increased residual urine, and spontaneous involuntary detrusor contraction (known as detrusor overactive) (Gray et al., 1995; Panicker et al., 2015). The most common clinical symptom of overactive detrusor reflexes is nocturia, followed by urge incontinence (Sakakibara et al., 1996a), characterized by a sudden difficulty in delaying urination. Sakakibara et al. (1999) reported that the incidence of detrusor hyperreflexia increased with WMLs in the same age group, while nocturia/urge incontinence was more associated with cognitive impairment than gait disorders, particularly in patients with WMHs at the top of the frontal horns of the lateral ventricles. One urodynamic study involving 106 patients investigating urinary incontinence after ischemic stroke suggested that 56% of patients had detrusor overactivity, 14% had detrusor overactivity with impaired contractility, 15% had detrusor underactivity, and 15% were healthy; follow-up of 63 patients at 1 month revealed that the prevalence changed to 48, 6, 16, and 30%, respectively (Pizzi et al., 2014). In another study, 9 patients with AD, 15 patients with mixed dementia (AD + WMLs), and 25 patients with VCI (WMLs) were assessed *via* cognitive scales [Mini-Mental State Examination (MMSE) and Alzheimer’s Disease Assessment Scale cognitive subscale], a urinary tract symptom questionnaire, and urodynamic studies (Takahashi et al., 2012). Patients with WMLs showed the best cognitive performance. Although the frequency of detrusor overactivity in urodynamic studies was similar between the groups, the sensation bladder volume was the lowest in WMLs, and increased daytime frequency, nocturia, and urinary incontinence were the most common in WMLs, suggesting WMLs contribute more to OAB and incontinence than AD in older adults with comorbid dementia (Takahashi et al., 2012). However, UD in VCI can be caused by detrusor overactivity as well as by mobility impairment, cognitive deficits, behavioral abnormalities, and urological problems (Panicker et al., 2015).

The progression of UD is variable; some stroke survivors experience acute onset of incontinence symptoms followed by a slow recovery (Williams et al., 2012; Thomas et al., 2019), while patients with cerebral small vessel disease may experience an insidious onset and slow deterioration, along with the gradual development of dysarthria, cognitive decline, and gait impairment. As the disease progresses, there is a vicious cycle of urinary incontinence and dementia (Skelly and Flint, 1995). UD onset varies in different types of cognitive impairment; incontinence tends to occur late in AD or Parkinson’s disease-related dementia and early in vascular dementia, frontotemporal dementia, Lewy body dementia, or normal pressure hydrocephalus (Panicker et al., 2015). Studies on nursing home residents have shown that patients with dementia who were continent upon admission were more likely to develop incontinence during the subsequent 12-month follow-up period, and incontinence symptoms were less likely to resolve in patients with cognitive impairment (Ouslander et al., 1993; Skelly and Flint, 1995). In studies of nocturia in community-dwelling older patients, the results of a multivariate analysis suggested that a higher MMSE score was a protective factor against nocturia in older men (Burgio et al., 2010; Lee et al., 2012). One review of eight cross-sectional studies suggested that nocturia and cognitive impairment are causal and share many risk factors (Haddad et al., 2020). Furthermore, in one clinical study of brain and urinary tract function in older patients with urinary incontinence, 128 patients with urinary incontinence (mean age, 79 years) and 27 continent controls were enrolled; 24-h voiding monitoring, cognitive function monitoring, video urodynamics, and single-photon emission computed tomography (SPECT) were performed (Griffiths, 1998). SPECT analysis revealed significant perfusion in the right superior frontal region and left global cortex of patients with urinary incontinence and reduced bladder filling sensation. Multivariate regression analysis revealed that urinary incontinence was associated with overall cognitive decline, especially temporal orientation (Griffiths, 1998). Presently, there are few studies on UD in VCI, and the correlation between the severity of cognitive impairment and UD should be further explored. Several clinical features may indicate UD in patients with VCI (Box 1), and the presence of multiple features may assist in efficient diagnosis.

**BOX 1 Clinical features of UD in VCI.**

• UD symptoms are mainly nocturia, urinary urgency, frequency, and urge incontinence
• History of stroke or multiple vascular risk factors, such as hypertension, diabetes, smoking
• Acutely progressive cognitive impairment
• Lower body parkinsonism
• Pseudobulbar palsy
• Pyramidal signs
• Predominantly male patients
• Combination of CSVD imaging markers such as WMHs, small subcortical infarcts, lacunes, CMBs, and PVSs

CMBs, cerebral microbleeds; CSVD, cerebral small vessel disease; PVSs, perivascular spaces; WMHs, white matter hyperintensities; UD, urinary dysfunction; VCI, vascular cognitive impairment.

## 4. Etiology and pathology

Vascular cognitive impairment is an umbrella term for cognitive disorders caused by vascular pathology, including atherosclerosis, demyelination, perivascular space enlargement, cerebral amyloid angiopathy, hypoperfusion, hemorrhage, and ischemic stroke, which often overlap with AD pathology (van der Flier et al., 2018; Iadecola et al., 2019). A meta-analysis of 19,040 participants from 36 prospective studies showed that WMHs increase the risk of both AD and vascular dementia, particularly in periventricular WMHs (Hu et al., 2021). One longitudinal community-based cohort study with 303 participants showed that WMH volumes were associated with secondary neurodegenerative changes, resulting in thinning of the rostral and caudal middle frontal cortex, paracentral cortex, and parietal regions, whereas parietal WMHs were associated with atrophy of the right frontal and left entorhinal cortices, which are related to decreased cognitive function (Rizvi et al., 2021). Consistent with these results, a recent meta-analysis and systematic review suggested that WMHs at baseline were associated with cognitive impairment in MCI and post-stroke populations with periventricular WMHs. Executive dysfunction was strongly associated with WMHs, particularly in the frontal region (Roseborough et al., 2022). Another meta-analysis of 2,950 patients from 12 cohorts showed that infarcts in the left frontotemporal lobes, right parietal lobe, and left thalamus were most closely associated with post-stroke cognitive impairment (PSCI) (Weaver et al., 2021).

**FIGURE 1:**
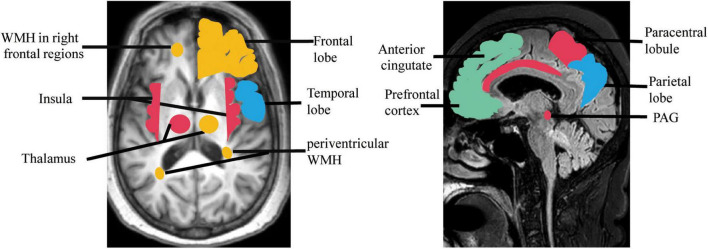
Regions showing brain damage in UD (red, green) and VCI (blue), and the overlapping regions (orange). PAG, periaqueductal gray; WMH, white matter hyperintensity.

To remain continent, a person first needs to recognize their need to urinate, then be able to tell their caregiver what they need and use a toilet (or use an appropriate appliance with help), and wait for the appropriate time to void. Dysphasia, aphasia, impaired mobility, and cognitive impairment are all related to urinary incontinence (Borrie et al., 1986; Brittain et al., 1998). Several factors contribute to the risk of incontinence in older adults, including urethral obstruction, urinary tract infections, sphincter injury, abnormal hormone levels, and polypharmacy (Hester et al., 2017; Lukacz et al., 2017). Both cerebral WMLs and brain damage are considered associated with UD (Sakakibara et al., 1996a,^1999^; Poggesi et al., 2008; Wakefield et al., 2010). In a longitudinal multinational study of 639 non-disabled older adults, severe age-related white matter changes were related to urinary urgency (Poggesi et al., 2008). Another study showed that WMHs in the right inferior frontal region and selected white matter tracts predicted incontinence onset, severity, and burden, and highlighted a role for the cingulum in bladder control (Kuchel et al., 2009). Other nearby white matter tracts such as the superior fronto-occipital fasciculus and anterior corona radiata may also be involved (Kuchel et al., 2009). A study on 72 patients with acute hemispheric stroke who provided UD histories and underwent urodynamic studies, showed that frontal and basal ganglia lesions were associated with hyperreflexia of the detrusor, frontal lobe injury was associated with loss of inhibition of sphincter relaxation, and basal ganglia lesions were associated with detrusor-sphincter desynchrony (Sakakibara et al., 1996a). A subsequent functional MRI study of 14 women with incontinence (aged >60 years) found associations between incontinence and activity of the rostral, posterior, and subgenual anterior cingulate gyrus; inferior frontal gyrus; orbitofrontal cortex; cuneus; parts of the parietotemporal lobe; insula; dorsal hippocampus; and parahippocampus (Tadic et al., 2010). The precise etiology and pathology of UD in VCI are unclear. However, some UD features overlap with those of VCI-related brain injury, which may account for the association between cognition and UD. Some evidence in men suggests that UD is an earlier predictor of vascular dementia than acute cerebral infarction (Kotsoris et al., 1987); however, it remains unclear if these lesions are coincidental or contribute to the observed clinical presentation (Figure 1). Further large-scale studies are required to elucidate these associations.

**FIGURE 2:**
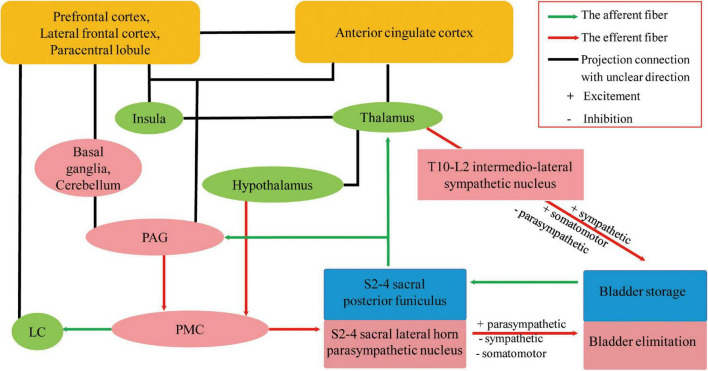
Possible neural circuits that coordinate urine storage and elimination (Fowler et al., 2008; Perneczky et al., 2008; Valentino et al., 2011; Griffiths, 2015; Hou et al., 2016; Merrill et al., 2016; Elman et al., 2021; Xiao et al., 2022). Part of the projection in the neural control of micturition is modified from Fowler et al. (2008), reproduced with permission from Springer Nature and Copyright Clearance Center. UD, urinary dysfunction; VCI, vascular cognitive impairment; PAG, periaqueductal gray; PMC, pontine micturition center; LC, locus coeruleus.

Many animal model studies have confirmed that normal urine storage and micturition require a complex neural network to coordinate activities of the urethral sphincters, urethra, and bladder (Fowler et al., 2008; Valentino et al., 2011; Hou et al., 2016; Merrill et al., 2016). Although the specific neural pathway remains unknown, it is now generally accepted that the spino-bubo-spinal reflex mediated by the pontine micturition center (PMC) is the basis of active urination control, and periaqueductal gray (PAG) is the switch that induces urination and maintains a strong connection to the prefrontal lobe (Griffiths, 2015). Fowler et al. (2008) employed transneuronal viral tracing in rats and found that the cerebral cortex, paraventricular nucleus, periventricular nucleus, medial preoptic area of the hypothalamus, PAG, PMC, locus coeruleus (LC) and subcoeruleus, red nucleus, raphe nuclei, and A5 noradrenergic cell groups were involved in the central control pathways of the bladder. Hou et al. (2016) showed that PMC neurons are adjacent to the LC in mice and project to the spinal cord while transmitting impulse signals to the LC, which transmits ongoing signals and regulates voiding by coordinating with forebrain regions (Valentino et al., 2011). In a study conducted by Sakakibara et al. (1996b), 39 patients with acute brainstem infarction were enrolled, and their micturitional history was collected. It was found that 49% of the patients had urinary irritation and obstruction symptoms within 3 months of cerebral infarction, the most common being dysuria; however, 28% had nocturia and 21% had urinary retention (Sakakibara et al., 1996b). A urodynamic study was performed on 11 patients with UD and 3 asymptomatic patients, from 1 day to 5 years after stroke. The results showed that 73% had detrusor overactivity and 9% showed low anterograde bladder; external sphincter electromyography was present in 45% of individuals, 45% had detrusor-sphincter dyssynergia, and 27% had uninhibited sphincter relaxation. In patients with UD, the lesions were mostly located in the dorsolateral pons including the LC, reticular formation, and pontine reticular nucleus, which were comparable to the PMC sites in animal experiments (Sakakibara et al., 1996b). Owing to the advancement in neuroimaging, functional MRI and positron emission tomography (PET) have allowed us to evaluate roles of the prefrontal cortex, anterior cingulate cortex, insula, hypothalamus, parahippocampal complex, supplementary motor area, PAG, and PMC in human bladder management (Griffiths, 2015; Xiao et al., 2022). One study using cranial PET for 44 patients showed that the right hemisphere, including the anterior cingulate cortex, parts of the premotor cortex, putamen, claustrum, and insula, plays an important role in patients with frontotemporal lobe degeneration and urinary incontinence (Perneczky et al., 2008). Considering current studies, we hypothesize that the central control of the bladder is as follows: as the urine volume increases, the bladder pressure increases, stimulating the bladder wall pressure sensors. Impulses not only travel to the sacral medullary cord but also to the PAG; when the PAG is activated, impulses are transmitted to the PMC, causing micturition. On the one hand, the PMC impulse afferents excite parasympathetic nerves, detrusor contraction, and internal urethral sphincter relaxation; on the other hand, they inhibit sympathetic nerves and external urethral sphincter relaxation and micturition occurs. PAG projects impulses down to PMC as well as to the prefrontal lobe, which interacts with other aforementioned brain regions to maintain normal urination, mainly by inhibiting the primordial urination reflex (Griffiths, 2015). The hypothalamus efferent projections go directly to the PMC, bypass the PAG, to send signals saying that it is safe or unsafe to void (Griffiths, 2015). Neural circuits joining the cortex to the lumbosacral spinal cord and to the bladder and urethra must remain intact for adults to urinate under suitable conditions (Xiao et al., 2022). These networks are shown in Figure 2.

In addition to the micturition reflex of parasympathetic nerve input, acetylcholine released by postganglionic cholinergic neurons acts on muscarinic acetylcholine receptors (mAChRs), influencing detrusor contraction and internal urethral sphincter relaxation. The T10-L2 spinal cord sympathetic nerves receive signals directly from the brain without going through the PAG, releasing norepinephrine that inhibits bladder body β-adrenergic receptors and excites the urethral sphincter and bladder neck α-adrenergic receptors (Griffiths, 2015). Therefore, parasympathetic stimulation promotes micturition, while sympathetic activation increases urine storage and suppresses urination, together to coordinate the micturition reflex. Cholinergic neurons have many important roles, ranging from more advanced functions such as learning, memory, attention, and sensorimotor processing to the maintenance of sleep–wake cycles and arousal (Lebois et al., 2018; Welk et al., 2021). The five mAChRs subtypes (M1–M5) are expressed throughout different regions of the body: M1, M4, and M5 subtypes are mainly expressed in the central nervous system; M2 in the heart; and M3 in the glands, bowel, and bladder (Welk et al., 2021), which are the basis for the adverse effects of the related drugs mentioned below. The LC is the major noradrenergic nuclei in the brainstem and is the primary source of intracranial norepinephrine, which is supplied to the forebrain to regulate behavior, cognition, and arousal (Elman et al., 2021) and not just part of PMC. A study involving 481 male patients who underwent neuromelanin-sensitive MRI revealed that the integrity of the LC is related to multiple cognitive domain functions, MCI, and daytime sleep-related dysfunction (Elman et al., 2021). A single-center, 12-month double-blinded crossover trial enrolled 39 cases of MCI and AD to assess biomarkers (Levey et al., 2022). The results revealed that atomoxetine, a selective noradrenaline reuptake inhibitor, significantly reduced cerebrospinal fluid total and phosphorylated Tau levels and normalized cerebrospinal fluid protein biomarker panels associated with brain metabolism, glial immunity, and synaptic function (Levey et al., 2022). In conclusion, the presence of UD in patients with VCI may be related to the impairment of the central autonomic pathway; however, whether LC impairment is involved requires further research.

## 5. Detection and evaluation

Many patients and caregivers do not report incontinence symptoms to physicians owing to neurological disorders such as aphasia, dysarthria, and cognitive impairment (Borrie et al., 1986; Brittain et al., 1998) or owing to personal embarrassment and misconceptions about treatment (Lukacz et al., 2017; Raju and Linder, 2020). Consequently, UD in patients with VCI is typically underdiagnosed. Urinary incontinence increases the risk of poor health outcomes and has been associated with the development of depression, which is detrimental to both the patient’s and caregiver’s quality of life (Henriksen, 1991). Importantly, screening for comorbidities (e.g., psychiatric-behavioral symptoms such as depression) and assessing how a patient’s quality of life is impacted are equally important.

**FIGURE 3:**
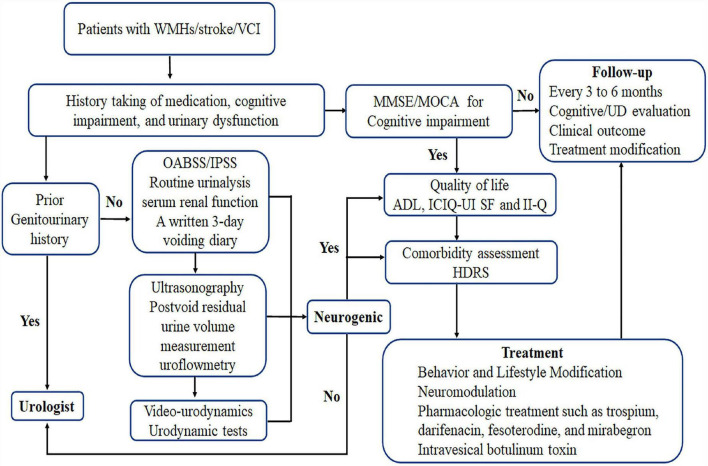
Assessment and treatment process and detection and evaluation process. OABSS, overactive bladder symptom score; IPSS, International Prostate Symptom Score; HDRS, Hamilton Depression Rating Scale; ADL, Activities Of Daily Living scale; ICIQ-UI SF, International Consultation on Incontinence Questionnaire-Urinary Incontinence Short Form; II-Q, Incontinence Impact Questionnaire; MMSE, Mini-Mental State Examination; MOCA, Montreal Cognitive Assessment; WMHs, white matter hyperintensities; UD, urinary dysfunction; VCI, vascular cognitive impairment.

Medical history is particularly important and should be taken for patients with UD combined with cognitive impairment, stroke, or imaging suggestive of intracranial WMLs, as well as for their caregivers. In the examination of present UD such as urinary frequency, urinary urgency, urinary incontinence, and nocturia, serum renal function and urinalysis should be used to identify urinary tract infection and glycosuria, a 3-day urinary diary to identify the type of voiding disorders (Bright et al., 2014; Groen et al., 2016), an overactive bladder symptom score to assess symptom severity (Homma et al., 2006), and a combined international prostate symptom score for male patients (Na and Cho, 2020) and a post-void residual urine examination *via* ultrasound and urinary flow rate evaluation to detect voiding disorders (Groen et al., 2016). Some patients with complex pathophysiology should undergo invasive urodynamics to assist in identifying underlying mechanisms (Panicker et al., 2015). The International Consultation on Incontinence Questionnaire-Urinary Incontinence Short Form (Avery et al., 2004; Abrams et al., 2006) and the Incontinence Impact Questionnaire (Homma et al., 2008) are valid and reliable tools that are suitable for the assessment of urinary incontinence impact on patients’ quality of life (Homma et al., 2008). In the examination of cognitive impairment, the MMSE and Montreal Cognitive Assessment scores can be used to evaluate general cognitive functions (van der Flier et al., 2018). The Activities Of Daily Living scale can help determine the severity of cognitive impairment (van der Flier et al., 2018) and the Hamilton Depression Rating Scale can be used to assess depression in patients with PSCI (Hamilton, 1960; Robinson and Jorge, 2016). Although the above scales have good clinical validity, they require further evaluation in patients with VCI and urinary tract symptoms. The International Consultation on Incontinence Questionnaire--Cognitively Impaired Elderly, a scale specifically designed to assess the impact of incontinence in patients with cognitive impairment, is currently under development^1^ (Abrams et al., 2006). The detection and evaluation process are shown in Figure 3.

## 6. Treatments

Patients with UD and VCI may have varying degrees of speech, motor, and cognitive impairment. Few studies have evaluated treatments for UD in patients with VCI, and although there is no robust evidence, behavioral therapy is the first choice for these patients, and for those who do not benefit from behavioral therapy, a combination of medications may improve symptoms (Panicker et al., 2015; Averbeck et al., 2017; Na and Cho, 2020). The prevalence of OAB increases with age, and the adverse effects associated with anticholinergic drugs used for OAB are more prevalent in VCI with blood–brain barrier disruption (Welk et al., 2021; Rajeev et al., 2022), especially in the presence of comorbidities and drug combinations (Na and Cho, 2020). Neuromodulation therapies without significant adverse effects have been shown to be effective for treating neurologically related lower urinary tract symptoms (Panicker et al., 2015). For more targeted treatment of UD patients with VCI, clinicians may assist in developing a treatment plan based on patient symptoms (nocturia, urinary urgency, frequency, stress, or urgency incontinence), patient and caregiver expectations, risk tolerance, and financial capacity.

### 6.1. Lifestyle and behavioral modifications

Lifestyle changes such as smoking cessation; limited water intake; weight loss; exercise; constipation treatment; and reduction of caffeine, carbonated drink, and alcohol intake are recommended for alleviating incontinence in older adults, without robust evidence (Hester et al., 2017; Lukacz et al., 2017). Indeed, it is unwise to restrict water intake in patients with post-stroke urinary incontinence (Thomas et al., 2019); a similar effect can be achieved by changing the way the patient drinks (e.g., changing from drinking large amounts of water at once to small, multiple drinks).

Behavioral modifications such as timed voiding, habit training, prompted voiding, pelvic floor muscle exercises, and biofeedback are all recommended for helping UD with VCI (Panicker et al., 2015; Averbeck et al., 2017; Na and Cho, 2020). Timed voiding involves voiding at regular intervals; habit training refers to an individualized voiding schedule based on the patient’s own pattern of voiding before incontinence; prompted voiding is when family members or professional caregivers regularly ask patients if they need to void and provide assistance when they go to the toilet; pelvic floor muscle exercises intentionally contract pelvic floor muscles to enhance inhibition of the detrusor muscle; biofeedback refers to measuring intravaginal pressure, actively or passively through electrical stimulation, to enhance pelvic floor muscle strength (Panicker et al., 2015; Na and Cho, 2020); and cognitive rehabilitation may also support patients with cognitive impairment (Lanctôt et al., 2020; Quinn et al., 2021). Timed voiding, habit training, pelvic floor muscle exercise, and biofeedback are for patients with mild VCI who can take care of themselves, while prompted voiding can be used for those with more severe dementia (Na and Cho, 2020).

### 6.2. Neuromodulation

A meta-analysis revealed that daily transcutaneous electrical nerve stimulation (TENS) and electroacupuncture at least 5 days per week starting less than 3 months post-stroke may be effective for treating urinary incontinence (Cruz et al., 2021). Another meta-analysis found that TENS may reduce the severity of urinary incontinence (Thomas et al., 2019) and that both TENS and neuromuscular electrical stimulation can reduce symptoms of post-stroke urge incontinence (Ali et al., 2022). One study showed that repetitive 5-Hz transcranial magnetic stimulation (rTMS) to the motor cortex was useful to facilitate the voiding phase in patients with multiple sclerosis (Centonze et al., 2007). In another study, a 2-week course of low-frequency rTMS (1 Hz) was effective in patients with Parkinson’s disease and UD, suggesting that rTMS could be effective in other neurological disorders with UD (Brusa et al., 2009). A prospective study of 10 patients with post-stroke cognitive impairment receiving 2 consecutive weeks (5 days per week) of high-frequency rTMS (20 Hz) to the ipsilateral dorsolateral prefrontal cortex showed that treatment improved cognitive function and changed brain networks associated with post-stroke cognitive impairment, which lasted at least 3 months (Cha et al., 2022). Although rTMS is now widely used for stroke survivors (Kim et al., 2020; Lefaucheur et al., 2020), there are no clinical studies related to rTMS in patients with UD, and VCI and further studies are required.

### 6.3. Pharmacologic treatments

Anticholinergic drugs can reduce bladder pressure by acting on antagonistic mAChRs, which are expressed throughout the bladder suburothelium, urothelium, and detrusor (Panicker et al., 2015). Since the Food and Drug Administration approved flavoxate and oxybutynin for treating OAB, several antimuscarinic agents such as solifenacin, fesoterodine, darifenacin, propantheline, tolterodine, propiverine, and trospium have been prescribed to patients with OAB (Madhuvrata et al., 2012; Mostafaei et al., 2020; Welk et al., 2021). Because M1–M5 mAChRs are expressed throughout the brain (Welk et al., 2021), the effects of anticholinergic drugs on cognitive impairment have garnered increasing attention. One study found that a 3-week oxybutynin regimen was equivalent to 10 years of cognitive aging (Kay et al., 2006) and should not be used for people with cognitive impairment. However, in both short- (1–3 weeks) and long-term (6–12 months) prospective studies, no effect of M3-specific anticholinergic drugs such as darifenacin on cognitive function was found nor that of the weak M3-selective solifenacin or the non-specific selectives trospium and fesoterodine (Welk et al., 2021). In contrast, observational studies have all found an association between anticholinergic drug use and cognitive decline (Pieper et al., 2020; Welk et al., 2021). However, the current study was not able to explain the causal relationship between anticholinergic drug use and cognitive impairment, and whether the so-called anticholinergic-induced cognitive impairment is reversible remains unclear (Welk et al., 2021) and limits the use of these drugs for patients with VCI. Trospium is plasma charged, and fesoterodine metabolites and darifenacin are macromolecules; they are all poorly lipophilic and hence do not easily cross the blood–brain barrier. If capable of improving the quality of a patient’s life, clinicians can choose a low-dose anticholinergic drug such as trospium, darifenacin, or fesoterodine with good physicochemical and clinical cognitive safety evidence and use it according to the patient’s condition and willingness (Welk et al., 2021). Meanwhile, β_3_-adrenoceptor agonists mirabegron and vibegron are effective in treating OAB and have not been associated with anticholinergic-related cognitive impairment (Escobar et al., 2021). Nevertheless, mirabegron may affect the cardiovascular system, inducing palpitations, facilitating or worsening hypertension, and rarely, inducing atrial fibrillation (Chapple et al., 2014; Escobar et al., 2021). Intradetrusor injection of botulinum toxin has shown to be effective and safe in neurogenic detrusor overactive incontinence (Panicker et al., 2015) and may also be superior to anticholinergic drugs in patients with dementia (Welk et al., 2021). These drugs should be prescribed after careful evaluation and consideration. A comprehensive assessment and treatment process are shown in Figure 3.

## 7. Conclusion

The review highlighted that WMLs, stroke, and VCI can interact with UD and that UD may be a clinical predictor of VCI. In the clinic, when patients present with WMLs, stroke, VCI, and UD, an accurate and timely evaluation of UD symptoms is required to treat modifiable factors. Lifestyle and behavioral modifications are the first treatment option for such patients, and those with poor results can also be treated with trospium, darifenacin, fesoterodine, and mirabegron according to the patient’s condition and willingness. Intravesical botulinum toxin and neuromodulation therapies, such as rTMS, may be promising. Additionally, to slow down the progression of VCI and reduce UD symptoms, management of cerebrovascular disease risk factors needs to be enhanced. This review also has some limitations. First, we only reviewed relevant articles in English; therefore, there may be some omissions. Second, there are few studies on this disease and most are reported by rehabilitation physicians and nurses, potentially introducing a biased perspective. Third, VCI is not a homogeneous disease but a spectrum of cognitive dysfunctions ranging from subjective cognitive impairment to MCI to dementia; UD is also a broad term used to encompasses a variety of urinary tract symptoms that are affected by many factors in older adults, such as various genitourinary tract diseases. However, in our clinical work, we found that several types of VCI are often combined with UD, causing distress to patients, with no disease-modifying treatment options for either VCI or combined UD, which is an urgent problem needing to be solved. In this review, we elaborated on the possible mechanisms and suggested that the noradrenergic LC may be involved; this may highlight a new horizon for in-depth studies to provide the basis for future drug development. Robust studies on the relationship between various symptoms of UD, the different degree of cognitive impairment, and related mechanisms are required to guide clinical practice.

## Author contributions

LSu conceptualized the study and revised the manuscript. PZ drafted and revised the manuscript. GZ and YS revised the manuscript and figures. YW, LSh, ZW, CW, and WZ reviewed the literature and revised the figures. All authors contributed to the writing and revisions of the manuscript and approved the final version.

## Footnotes

1
https://iciq.net/modules
